# Acute/Chronic Responses of Combined Training on Serum Pro-thermogenic/Anti-inflammatory Inducers and Its Relation With Fed and Fasting State in Overweight Type 2 Diabetic Individuals

**DOI:** 10.3389/fphys.2021.736244

**Published:** 2022-01-20

**Authors:** Ivan Luiz Padilha Bonfante, Renata Garbellini Duft, Keryma Chaves da Silva Mateus, Joice Cristina dos Santos Trombeta, Enrico Antonio Rautenberg Finardi, Ana Paula Boito Ramkrapes, Diego Trevisan Brunelli, Marcelo Alves da Silva Mori, Mara Patricia Traina Chacon-Mikahil, Licio Augusto Velloso, Cláudia Regina Cavaglieri

**Affiliations:** ^1^Laboratory of Exercise Physiology, Faculty of Physical Education, University of Campinas, Campinas, Brazil; ^2^Federal Institute of Education, Science and Technology of São Paulo, Hortolândia Campus, Hortolândia, Brazil; ^3^Laboratory of Aging Biology, Department of Biochemistry and Tissue Biology, University of Campinas, Campinas, Brazil; ^4^Obesity and Comorbidities Research Center, University of Campinas, Campinas, Brazil; ^5^Laboratory of Cell Signaling, Department of Internal Medicine, University of Campinas, Campinas, Brazil

**Keywords:** brown adipose tissue (BAT), browning of white adipose tissue (WAT), inflammation, interleukins, metabolism, myokines, type 2 diabetes

## Abstract

Concentrations of pro-thermogenic/anti-inflammatory inductors are influenced by fed/fasting, sedentary/trained states, and metabolic pattern. However, there is a lack of information on the interactions of these conditions, especially in humans. Thus, the present study aimed to evaluate the chronic and acute training responses as well as the fed/fasted states of serum pro-thermogenic/anti-inflammatory inducers in overweight type 2 diabetics individuals. Fifteen individuals with type 2 diabetes [body mass index (BMI): 29.61 ± 3.60 kg/m^2^; age: 50.67 ± 3.97 years] participated in the study. In the pre- and post-experimental periods, baseline clinical parameters analyses were performed. Pro-thermogenic/anti-inflammatory inductors were evaluated pre/post-baseline and before, shortly after, and after 30′ and 60′ in the first and last sessions of a 16-week combined training (CT) period. These inducers were also compared for fasting and feeding before and after the training period. CT has improved baseline physical fitness, metabolic pattern, and it has also increased interleukin (IL)33 and FNDC5/irisin. In the first training session, there was a decrease in IL4, IL13, and IL33, besides an increase in FNDC5/irisin, and natriuretic peptides. In the last training session, there was an increase in natriuretic peptides and bone morphogenic protein 4 (BMP4). Differences in responses between the first and last training sessions were observed at certain post-session times for IL4, IL33, and natriuretic peptides, always with higher concentrations occurring in the last session. In evaluating the area under the curve (AUC) of the first and last training session, FNDC5/irisin, natriuretics peptides, and meteorin-like showed increased areas in the last training session. The pre-training fed state showed an increase in IL4 and IL33, while in fasting there was an increase in meteorin-like, natriuretic peptides, and FNDC5/irisin. In the post-training, IL4, IL13, and IL33 were increased in the fed state, while meteorin-like, natriuretic peptides, and FNDC5/irisin remained increased in the fast. Adaptation to physical training and a better metabolic pattern favor an improvement in the acute secretory pattern in part of pro-thermogenic and anti-inflammatory substances analyzed. The fed and fasting states also interfere differently in these substances, where fasting interferes with the increase of myokines, while the fed state induces an increase in interleukins.

**Clinical Trial Registration:** [http://www.ensaiosclinicos.gov.br/rg/RBR-62n5qn/], identifier [U1111-1202-1476].

## Introduction

The induction of adipocyte thermogenesis is effective to treat cardiometabolic diseases such as obesity and type 2 diabetes (T2D) since it is associated with increased energy expenditure, weight loss, improved insulin sensitivity, and glucose uptake (Villarroya and Vidal-Puig, 2013).

Physical exercises and certain dietary states/patterns are physiological strategies that show evidence of the thermogenic and mitochondrial capacity of adipocytes stimulation *via* sympathetic or hormonal signaling mediated by serum pro-thermogenic products that are also associated with cardioprotective and anti-inflammatory effects (Bostrom et al., 2012; Villarroya and Vidal-Puig, 2013; Fabbiano et al., 2018).

The stimulus for the secretion of these pro-thermogenic products induced by physical exercise or dietary state/pattern is due to factors as energy need, hypoxia, and metabolic/thermal stress, which stimulate the secretion of catecholamines and other pro-thermogenic substances such as interleukins 4 (IL4), IL13 and IL33, fibroblast growth factor 21 (FGF21), irisin, natriuretic peptides, meteorin-like and bone morphogenic protein 4 (BMP4), and BMP7 (Tseng et al., 2008; Schulz et al., 2013; Qian et al., 2013; Kajimura and Saito, 2014; Lee et al., 2014; Rao et al., 2014; Brestoff et al., 2015; Li et al., 2015; Modica et al., 2016; Hoffmann et al., 2017). However, most of these findings related to pro-thermogenic factors are based on experimental studies with animal or *in vitro* models, not yet been comprehensively evaluated in humans, even more so with the interaction between physical exercise, dietary status, and worsened metabolic state like T2D.

Certain studies show that in physical exercises the effects are not consensual on some of these pro-thermogenic factors, which seems to vary according to the moment of the analysis (right after the practice or baseline) and the level of physical fitness of the analyzed subject (sedentary versus trained) (Huh et al., 2012, Huh et al., 2014; Petriz et al., 2017). A single bout of low exercise intensity already induces at least moderate favorable changes in glycemic and lipidemic profiles after a certain breakfast pattern, for example (Benedini et al., 2019). In addition, certain catabolic and inflammatory markers showed differences between acute and chronic responses to functional and resistance exercises (Faelli et al., 2020). In food, factors such as the postprandial period (Din et al., 2018), caloric restriction/fasting (Roca-Rivada et al., 2013; Fabbiano et al., 2016, 2018) also appear to influence the secretion of some of these substances and products related to the energy balance, especially in the short term (Benedini et al., 2011).

Other factors that seem to influence the combination of these markers are the metabolic pattern, the presence of overweight, T2D, and insulin resistance, for example, which is associated with lower baseline concentration of some of these pro-thermogenic factors such as irisin or natriuretic peptides (Du et al., 2016; Verboven et al., 2017). These effects are probably related to the sedentary lifestyle and positive energy balance present in these diseases, which decreases the secretion of pro-thermogenic/anti-inflammatory products, especially myokines (Gleeson et al., 2011; Eckardt et al., 2014). Additionally, these chronic diseases are associated with low-grade chronic inflammation, characterized by increased production of inflammatory inducers and decreased anti-inflammatory products (Gleeson et al., 2011).

Based on the pro-thermogenic function and anti-inflammatory effects of these cited substances, in addition to the lack of information related to the influence of the fed/fasting, sedentary/trained states and metabolic pattern on its concentrations, as well as the interaction between these conditions in humans, the aim of the present study was to evaluate the acute/chronic effects of training on pro-thermogenic/anti-inflammatory serum inducers in type 2 overweight diabetics and the influence of the fed/fasting state on these markers. We hypothesized that an exercise session could induce increases in the pro-thermogenic/anti-inflammatory serum inducers, but that metabolic and physical fitness improvement provided by the adaptation to physical training would induce an even greater increase in the secretion of these products. In addition, we also assume that fasting and being fed in different ways influence these pro-thermogenic/anti-inflammatory products according to their characteristics (cytokines × myokines) and that adaptation to training could mitigate these differences.

## Materials and Methods

### Volunteers

This study presents the secondary results (acute training and fed/fasted state responses on pro-thermogenic/anti-inflammatory; and the association between these results and basal metabolic/clinical markers) from the randomized controlled trial UTN: U1111-1202-1476^1^. The primary results (molecular and thermogenic profile of adipocytes; clinical and thermogenic basal markers) were presented in a randomized controlled trial (Bonfante et al., under review). The study was approved by the Research Ethics Committee of the University of Campinas Medical School and was based on the principles of the Declaration of Helsinki.

The study inclusion criteria were: diagnosis of T2D; age between 40 and 60 years old, body mass index (BMI) from 25 to 35 kg/m^2^; non-active life habit; not having regularly participated in any training program and/or executed any systematized diet during the previous 12 months of study; to have availability to participate in the first and last training sessions protocols. The exclusion criteria were: arrhythmia or cardiac ischemia, coronary artery disease, severe arterial hypertension, chronic obstructive pulmonary disease, anemia, uncontrolled hypothyroidism, limiting bone-joint diseases; general and or specific serious complications of T2D; not be approved in exercise electrocardiogram; to use some medicine as exogenous insulin, thiazolidinediones, beta-blockers, anticoagulants, anti-inflammatories. The medicines used are shown in Table 1. No changes were made in the medicines used (types and dosages) during the experimental period. As criteria for discontinuity, were adopted: the lack of motivation or willingness of the volunteer to attend training sessions; attendance at training sessions below 85% and/or more than three consecutive absences; other risks that could occur to the volunteers even after clinical release.

**TABLE 1:** Medicine used by subjects.

	Number of subjects using the medicine
Sulfonylurea	6
Metformin	15
SGLT2 blockers	2
DPP-4 inhibitors	3
Lipid-lowering drug therapy	5
Antihypertensive	8
Antidepressant	2
Levothyroxine	2
Vitamin D	1
Calcium	1

*SGLT2, sodium-glucose cotransporter 2; DPP4, dipeptidyl peptidase-4.*

**FIGURE 1:**
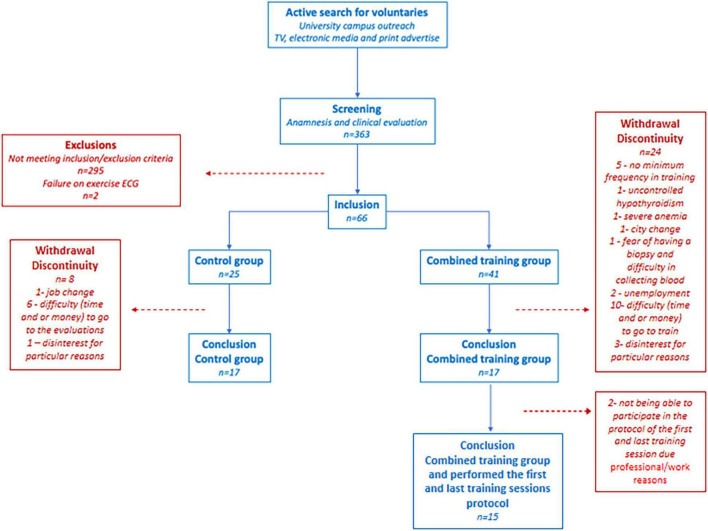
Flowchart.

The clinical and biochemical data have already been presented in the primary outcomes of the trial, with a comparative analysis between-group (training and control) versus time and a total of 17 subjects per group. All subjects were recruited through publicity in the media and university. Recruitment was set to the primary and secondary objectives of the research project. In the first round, 363 volunteers were screened; 297 were excluded due to the inclusion/exclusion criteria. The 66 volunteers included were randomly assigned to the combined training group (CTG) (*n* = 41) or control group (CG) (*n* = 25). More subjects were included in the CTG due to the greater risk of discontinuation. The randomization number sequence was created using Excel 2019 (Microsoft, Redmond, WA, United States) with a 1:1 ratio allocation. The randomization was performed by a researcher not involved in this project to avoid selection bias. During experimental protocol time (training or control), 30 volunteers were excluded due to failure to adhere to the protocol (job change, city change, difficulty to go to the evaluations, and/or to train for lack of time or money and other particular reasons). Another two volunteers were excluded for medical reasons, uncontrolled hypothyroidism and anemia, identified in laboratory tests. Thus, 34 volunteers completed the study, 17 in the CTG (10 women and 7 men) and 17 in the CG (8 women and 9 men). Of the 17 volunteers in the CTG, 15 (9 women/6 men) underwent first and last training sessions protocols in order to present training adaptations and differences between sedentary and trained states. The complete flowchart is shown in Figure 1. The reason not all participants underwent first and last training sessions was the time limitation of the volunteers because of professional/work reasons. In addition, due to technical limitation (error in performing two enzyme-linked immunosorbent assay (ELISA) kit plates, one of FNDC5/irisin and one of the natriuretic peptides), we had an “*n*” of 8 subjects (4 men and 4 women) for FNDC5/irisin and an “*n*” of 12 individuals (5 men and 7 women) for natriuretic peptides. A similar male to female ratio was maintained for FNDC5/irisin and natriuretic peptides according to the distribution of our samples on the analysis plates.

A power analysis was conducted *a priori* and *a posteriori* with G*POWER 3.1 software (Universitat Kiel, Germany). *A priori*, we based initially on waist circumference, since the amount of abdominal fat is one of the main factors related to T2D and interfere with primary outcomes analyzed in the first study. We based on a study that aimed to analyze the combined training effects in middle-aged subjects, type 2 diabetics, which presented the following waist values in (cm): (pre 118.6 ± 11.6 and after 112.6 ± 11.9) (Lucotti et al., 2011). A total of 28 participants was obtained (14 per group, training versus control), considering a statistical design of the *F* test (2 × 2 ANOVA for repeated measures), calculation of the *f* effect size of 0.53, type I error (α) 5%, a correlation coefficient of 0.5, and test power size of at least 95%. Additionally, for the present study design, we based pre–post acute effects of exercise on irisin levels from a study with healthy young men, who performed one session of endurance and another of resistance exercise (Nygaard et al., 2015). Although we analyzed several markers in the first and last training sessions, we chose irisin because it is one of the main circulating inducers associated with exercise, thermogenesis, and metabolic health (Bostrom et al., 2012; Lee et al., 2014; Otero-Diaz et al., 2018). The study used presented the following irisin values in (ng/ml): (endurance exercise pre 382 ± 41 and after 459 ± 51; resistance exercise pre 355 ± 50 and after 437 ± 56) (Nygaard et al., 2015). A total of six subjects would be needed based on endurance exercise and eight subjects based on resistance exercise, considering: a statistical design of the *F* test (one-way ANOVA for repeated measures, within factors); calculation of the *f* effect size = 1.66 (endurance exercise) and 1.54 (resistance exercise); type I error (α) 5%, correlation coefficient of 0.5; and test power size of at least 95%. Still using this data of Nygaard et al., 2015, however, for a statistical design of the *F* test (2 × 2 ANOVA for repeated measures), the results were a sample of six subjects per group (first session group versus last session group) based on both, resistance and endurance data. Finally, we calculated the posteriori power of FNDC5/irisin results from eight participants (first training session). This calculation was based on the statistical test used in the study, an alpha significance level of 0.05, an effect size of 0.75 from the pre and post 60′ values (post 60′ was the higher value of time course) of the means and standard deviation of FNDC5/irisin levels. As a result, we observed a beta power of 0.998 for a statistical design of the *F* test (2 × 2 ANOVA for repeated measures) and 0.997 (one-way ANOVA for repeated measures, within factors). These beta powers is up from the normally stipulated power of 0.80, which is usually used as a minimum required to characterize a sample as capable of detecting a difference in a given population.

### Experimental Design

After a media call by volunteers, structured anamnesis and physical activity questionnaires were applied to those interested in participating in the study to meet the volunteers and evaluate the inclusion and exclusion criteria. Those who met the inclusion criteria underwent clinical and medical evaluations.

Those approved to participate were familiarized with the location, tests, and equipment used from visiting the premises where the assessments were carried out, as well as explaining and experiencing the procedures and tests that would be used.

Clinical evaluation, functional evaluation tests, collection of biological material, and training practice were performed under conditions of spontaneous breathing of atmospheric air in a room with an average ambient temperature of 23°.

**FIGURE 2:**
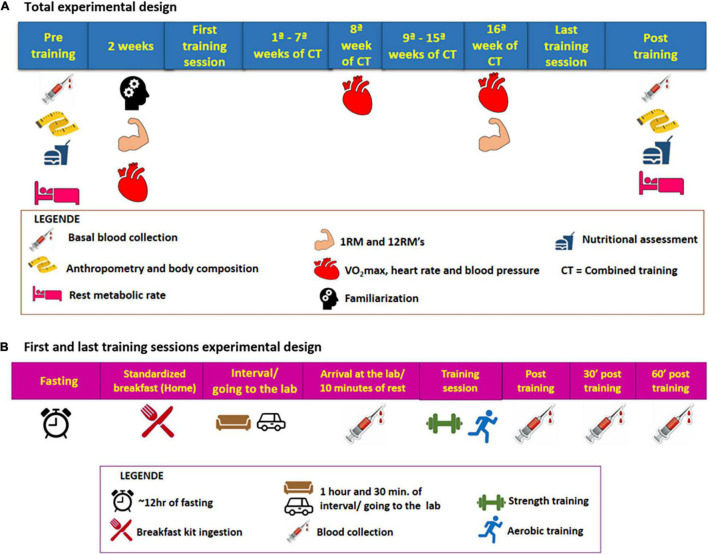
**(A)** Total experimental design (chronic design). **(B)** First and last training sessions experimental design (acute design).

Initially, the subjects underwent baseline assessments: anthropometric, body composition, functional [cardiorespiratory, muscle strength, resting metabolic rate (RMR)], nutritional assessment, blood collection, and hemodynamic. All of these initial assessments lasted for around 2 weeks, always with an interval between 48 and 72 h between assessments that required abstinence from physical exercises or previous stress tests. Also, it was recommended before each assessment to abstain from alcohol for 24 h, caffeine, or any stimulating drink for 12 h. For blood collection and RMR, fasting was still recommended for 12 h. All assessments were repeated after 16 weeks of training following the same initial recommendations. Before the last training session in the 16th week of training, VO_2_max and 12-maximum repetition tests were performed. The participants underwent two sessions of familiarization with the equipment and training protocol before strength assessments. The experimental design is detailed in Figure 2A.

After these baseline evaluations, participants performed the pre-training session, after having a standard breakfast (20 g of Quaker^®^ oats (Quaker Brazil, Guarulhos, Brazi), 1 pot of 90 g Nestlé^®^ light Greek yogurt (Nestlé Brazil, São Paulo, Brazil), 1 packet of whole Nesfit^®^ cookies (Nestlé Brazil, São Paulo, Brazil) and banana) in their homes after 10–12 h of fasting. To carry out this pre-session, the subjects followed the following recommendations: take the medication normally; wear appropriate clothes to exercise; eat only a standard breakfast 1 h and 30 min before the experimental session and; abstain from caffeine or any stimulating drink for 12 h and alcohol for 24 h and no exercise during the 48–72 h before the session. The volunteers were instructed to make a note of their previous dinner and try to repeat it in the last-training session. Before (after 10 min sitting at rest), immediately after, 30 min and 1 h right after the session, blood samples were taken using an intravenous catheter. The catheter was inserted before the training session and remained throughout the exercise session and in the resting period after the end of the exercise. The entire procedure followed before and during the first session was performed again in the last training session. For more details vide Figure 2B. The post-session blood collection times were based on time course studies of irisin secretion by exercises and on the idea that the analyzed analyte secretion is stimulated by the energy needed. This entire protocol was repeated in the last training session. During the pre- and post-training sessions and in the subsequent resting period, the participants could use the bathroom and hydrate by drinking water freely.

### Evaluation Protocols

#### Physical Activity Level and Exercise Electrocardiogram

Baecke and IPAQ questionnaires were applied to assess the level of habitual physical activity of volunteers (Nyssen et al., 2013). The questionnaires showed that the volunteers did not perform systematic physical activity in the period before the study and had an average weekly physical activity time of 105.50 ± 66.03 min. Volunteers who met the inclusion criteria underwent an exercise electrocardiogram (Bruce protocol) (Brunelli et al., 2015).

#### Anthropometry and Body Composition

Bodyweight and height were measured on a platform scale (Filizolla^®^, São Paulo, Brazil) with a coupled stadiometer. From the weight and height data, BMI was calculated. Neck, waist, and hip circumferences were measured using a measuring tape with an accuracy of 1 mm and based on standard anatomical references for these regions.

Body composition was assessed by whole-body plethysmography (air displacement plethysmography, BOD POD*^R^* – COSMED USA, Inc., Concord, CA, United States), based on the criteria described in the equipment manual and previously used (Gomez-Ambrosi et al., 2011).

#### Muscle Strength

After familiarization, the 12-maximum repetition (adapted) test was performed to determine the load of each exercise used in the strength training protocol (Abdul-Hameed et al., 2012). Three to 6 attempts were made, with a 2- to 3-min interval to obtain the 12 maximum repetitions.

The maximum repetition test (1RM) was also applied (Brunelli et al., 2015), which consisted of assessing the strength of the lower and upper limbs using the leg press and bench press exercises, respectively.

In the pre-trial period, this test was performed twice (test and re-test), the first being used to familiarize and minimize the influence of neural adaptations gains and the learning effect, while the second was used as a reference value. At the post, there was only one re-evaluation.

#### Cardiorespiratory Assessment, Heart Rate, and Blood Pressure

Baseline heart rate and blood pressure were measured after 5 min of rest (seated), using a cardio frequency meter and a mercury sphygmomanometer and stethoscope. The cardiorespiratory assessment was performed using a progressive effort protocol on a treadmill (Quinton, model TM55, United States), with a continuous collection of expired gases breath to breath (CPX Ultima, MedGraphics, United States). The cardiorespiratory fitness verified by the maximum oxygen consumption (VO_2_max) was determined by the average values of the last 30 s of the test. The criteria used for stopping the cardiorespiratory assessment were based on the perceived exertion equal to 20 (Borg scale 6–20), Respiratory Exchange Ratio (RER > 1.1), maximum heart rate within 10 beats of the age-appropriate reference value, and inability to maintain the speed on a treadmill (Brunelli et al., 2015).

#### Rest Metabolic Rate

The determination of the RMR was performed using oxygen consumption and carbon dioxide production, using indirect open circuit calorimetry by the gas analysis system (Ultima CPX, MedGraphics, United States) which was calculated in daily values (kcal/day) by the Weir equation (Bonfante et al., 2017).

For this test, the volunteers wore a face mask connected to the gas analyzer, remaining silent, in the supine position, avoiding moving and not sleeping for 30 min, so that breath by breath was captured. The gas analyzer was calibrated before each test. The first 10 min were not considered for RMR calculation, because of stabilization of the physiological variables at resting state. The last 5 min also were not considered due to the evaluator’s movement in the room. In addition, we oriented the participants to make their locomotion by motor vehicle to the testing site to ensure minimal physical activity before RMR determination.

#### Blood Samples

Blood samples were collected at baseline-post 16-week experimental protocol, and in first and last training sessions (before, immediately after, post 30′, post 60′ of training sessions). For the food status, the baseline-post 16-week experimental protocol was used to assess the influence of fasting, while the moment pre of first and last sessions’ were utilized to characterize the feeding pattern.

Baseline blood samples (approximately 40 ml) were collected from the antecubital vein in a dry vacuum tube with anticoagulant (EDTA). All of them were always performed at the same time (between 7:00 and 9:00 am), after a period of abstinence from exercising between 48–72 and a 12-h fast.

The collections of the training sessions (between 7:00 am and 10:00 am), 2 h after eating a standardized breakfast. Blood samples were collected in one dry vacuum tube and one tube with anticoagulant (EDTA) (one tube each for the time of collection). Both blood collections were performed using an intravenous catheter and a PRN adapter plug inserted into the brachial vein at the height of the antecubital fossa, which remained throughout the exercise session and in the posterior rest period.

Some samples were readily analyzed and others were stored in a −80° freezer after being centrifuged for further analysis.

#### Biochemical Analyses

The plasma glucose analysis was performed in the GOD-Trinder method. The serum was used to analyze: insulin, C peptide, TSH (chemiluminescence method), triglycerides, total cholesterol (enzyme-trind method), high-density lipoprotein (HDL) (accelerator-selective detergent method), very low-density lipoprotein (VLDL), and low-intensity lipoprotein (LDL) (Friedewald formula method). Using whole blood were made glycated hemoglobin (high-performance liquid chromatography method) and blood cell counts (automated microscopy method). From the results of glucose and Insulin, the HOMA-IR index was calculated using the formula (Bonfante et al., 2017).

The concentrations of cardiac natriuretic peptides (*Aviva Biosystems*, United States) IL4, IL13, IL33, FGF 21, BMP4, BMP7 meteorin-like (*R&D System*, United States), and FNDC5/irisin (*US Biological*, United States) were performed by the method ELISA according to the manufacturers’ specifications.

#### Nutritional Assessment

The volunteers were instructed to maintain their eating habits throughout the study. To ensure that this recommendation was being followed, the volunteers were routinely alerted to this aspect and even filled out food records (FRs) before and after the experimental period. They were asked to report in the FR all the food and drinks eaten during the established days (different and non-consecutive days; two weekdays and one weekend day to have an average of the three recalls) (Brunelli et al., 2015).

The FR was analyzed semi-quantitatively delimiting the number of portions ingested per day of carbohydrates, raw and cooked vegetables, fruits, milk and dairy products, meat, legumes, and energy supplements. Subsequently, these portions had their amounts of macronutrients (in grams) calculated and the total caloric intake, based on the pre-established portions and formulas (Brunelli et al., 2015).

#### Combined Training Protocol

The 16 weeks of combined training consisted of sessions with 5 min warm-up on an exercise bike and subsequent performance of the resistance training (RT), followed by the aerobic training (AT) in the same session, divided into two stages and performed on three alternate days a week (Mondays, Wednesdays, and Fridays). The entire volume and intensity of the training followed the recommendations for subjects with T2D (Colberg et al., 2010, 2016).

In stage 1, the RT was composed of a linear periodization with 10 exercises (7–8 per day) with priority to work large muscle groups, which are: leg press, knee extension, knee flexion, bench press, high pull (performed in three training days off a week) and barbell curls, triceps pulley, smith machine shoulder press, extension/flexion of the calf and upper abdominal (performed one or two training days a week). These exercises had 3 sets of 12 submaximal repetitions and a 1-min pause between sets, which took approximately 37–42 min to perform the RT. The ordering of the exercises was alternated by segment in this phase. Then the participants moved to the treadmill and occasionally to the athletics track, where they performed 35 min of AT with walking/trotting/running (according to the volunteer’s physical fitness level in the VO_2_max test), with intensity variation between 50 and 65% of VO_2_max., being 3.5 min at 50–55% of VO_2_max., 14 min at 55–60% of VO_2_max., 14 min at 60–65% of VO_2_max., and 3.5 min between 50 and 55% VO_2_max (Colberg et al., 2010, 2016).

In stage 2, the RT session was performed with the same exercises and series as stage 1; however, with 10 submaximal repetitions and a 1-min and 15-s pause, totaling approximately the same 39–42 min as stage 1. This was done to maximize the load increase by the submaximal repetition test based on the principle of interrelation between volume/intensity. With the decrease in volume, there was an increase in the load when compared to the previous stage. In this stage, the ordering of the exercises was performed by the initial performance of the upper muscle segment, followed by the lower or vice versa (it is recommended to alternate the initial segment at each session). For AT there was the same training pattern, but there was an adjustment in the training intensity zones (a new VO_2_max test was performed after the 8th training week). AT intensities were between 50 and 70% of VO_2_max, with 3.5 min at 50–60% of VO_2_max, 14 min at 60–65% of VO_2_max., 14 min at 65–70% of VO_2_max., and 3.5 min between 50 and 60% VO_2_max (Colberg et al., 2010, 2016).

Throughout the program, the intensity of the AT was controlled by the speed associated with the percentage of VO_2_max. predicted provided at each stage of training, besides verification of heart rate and subjective perception of effort (Borg scale).

Regarding adaptation to AT (decreased HR and or perceived exertion below or equal to “relatively easy/easy”) before the change in training intensity by the intermediate aerobic reassessment of 8 weeks or during any training period, 0.2 km/h were increased at each intensity of aerobic exercise.

In the RT, load readjustments were carried out every 2 weeks in the first month and then weekly, with the application of submaximal repetition tests. With this procedure, it was observed a progressive increase in training overload and loads that were for most volunteers between 50 and 75% of 1RM (Colberg et al., 2010, 2016).

#### First and Last Training Sessions

In the first and last sessions of the training program, the general characteristics of the proposed Combined training (CT) were followed with some modifications to avoid major overload in the pre-session by the untraining volunteers at this time. In RT, the priority of working in large muscle groups was maintained (seven exercises), which were performed by 2 sets of 12 adapted submaximal, a pause of 1 min between series and alternating exercise order by segment and execution speed of 2 s for the eccentric movement and 2 s in the concentric movement, having a total duration of approximately 25 min. Afterward, 25 min of AT (walking/trotting/running) was performed on a treadmill alternating the intensity by 5 min at 40–45% of the VO_2_max., 7.5 min at 45–50% of the VO_2_max., 7.5 min at 50–55% of the VO_2_max., and 5 min between 40 and 45% of the VO_2_max. Before the start of the session, a 5-min warm-up was performed on an exercise bike. The same characteristics of the first training session were maintained in the last session, however, the intensity of the RT and AT were adjusted according to the results of the previous VO_2_max and 12-maximum repetition tests, which were performed pre-trial period and before the last training session. The sessions were supervised using the BORG scale and the heart rate monitor (Colberg et al., 2010, 2016).

#### Statistical Analysis

Initially, the Kolmogorov–Smirnov test was applied to test data normality. In the case of non-parametric data, transformation into log Ln was applied.

For the comparison between the data of the pre and post in the same condition (baseline clinical and biochemical parameters; pro-thermogenic/anti-inflammatory inducers in food status), Student’s *t*-test was applied for dependent samples.

Student’s dependent *t*-test was used to compare pre- and post-training moments in isolation of fed versus fasting state. To compare pre- and post-training food status together (pre–post fasting versus pre–post fed) was applied repeated measures two-way ANOVA or analysis of covariance (ANCOVA). In ANOVA two-way, whenever a significant *F* value was obtained (group × time significant interaction value “*P*”), Tukey’s *post hoc* test was applied.

In training sessions results, primarily, repeated measures ANOVA (one-way) was applied to compare the time effect separately in the first and last training sessions. Whenever a significant *F* value was obtained in ANOVA one-way (time significant value “*P*”), Tukey’s *post hoc* test also was applied. Posteriorly, repeated measures ANOVA two-way and Tukey’s *post hoc* (if applicable) were performed to determine significant differences in group × time between first and last training sessions comparisons. In addition, the area under the curve (AUC) was calculated by trapezoidal approximation for each analyte analyzed in the first and last training session. The comparison between the AUC of the first and last training sessions was made by student’s *t*-test.

Pearson’s correlation coefficient was also applied to assess the correlation between Δ% basal serum pro-thermogenic/anti-inflammatory inducers and clinical variables.

**FIGURE 3:**
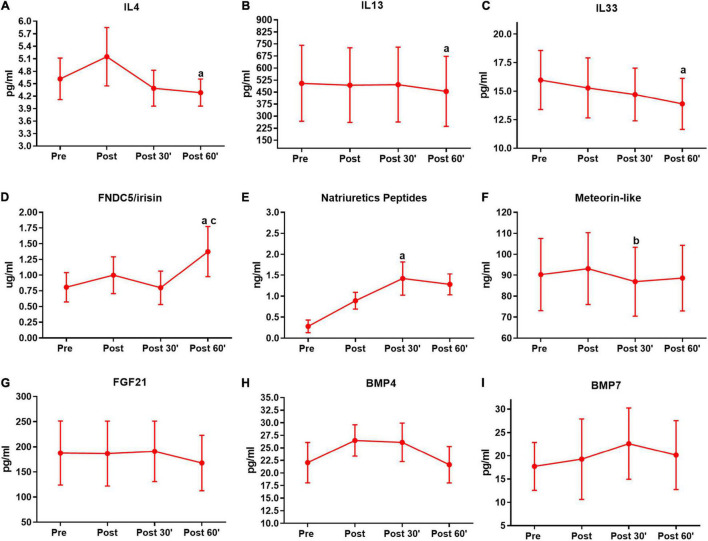
Serum pro-thermogenic/anti-inflammatory inductors time-course in the first training session. **(A)** Interleukin 4 (IL4). **(B)** IL 13. **(C)** IL33. **(D)** FNDC5/irisin. **(E)** Natriuretic peptides. **(F)** Meteorin-like. **(G)** Fibroblast growth factor 21 (FGF21). **(H)** Bone morphogenic protein 4 (BMP4). **(I)** BMP7. ^a^*P* < 0.05, difference from the moment pre. ^b^*P* < 0.05, difference form the moment post. ^c^*P* < 0.05, difference from the moment 30′. *n* = 15 per group, except FNDC5/irisin *n* = 8; and natriuretic peptides *n* = 12, both in feeding group. Values are in mean ± standard error.

**FIGURE 4:**
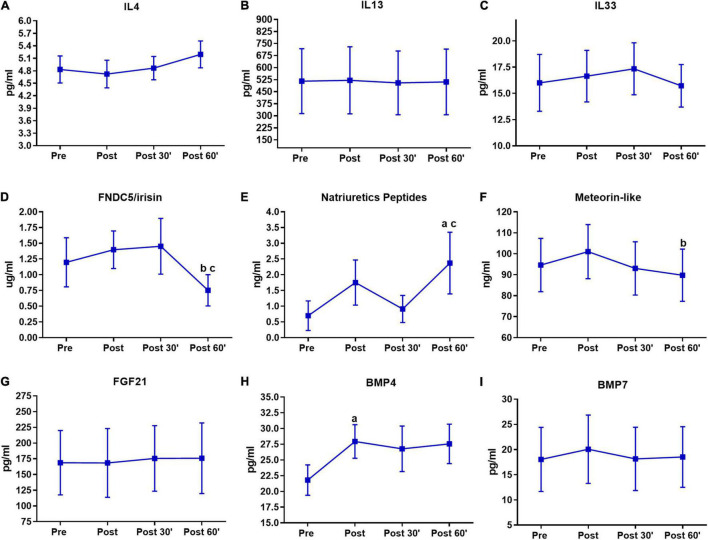
Serum pro-thermogenic/anti-inflammatory inductors time-course in the last training session. **(A)** IL4. **(B)** IL 13. **(C)** IL33. **(D)** FNDC5/irisin. **(E)** Natriuretic peptides. **(F)** Meteorin-like. **(G)** FGF21. **(H)** BMP4. **(I)** BMP7. ^a^*P* < 0.05, difference from the moment pre. ^b^*P* < 0.05, difference form the moment post. ^c^*P* < 0.05, difference from the moment 30′. *n* = 15 per group, except FNDC5/irisin *n* = 8; and natriuretic peptides *n* = 12, both in feeding group. Values are in mean ± standard error.

**FIGURE 5:**
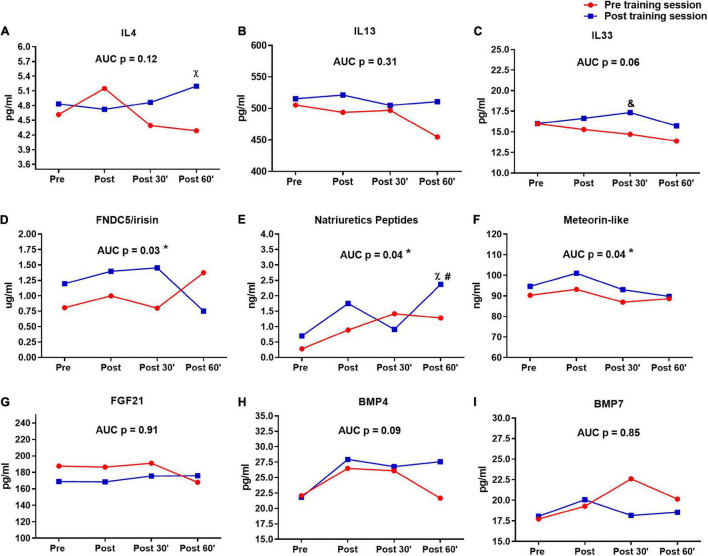
Serum pro-thermogenic/anti-inflammatory inductors time-course in the pre- and post-training sessions. **(A)** IL4. **(B)** IL 13. **(C)** IL33. **(D)** FNDC5/irisin. **(E)** Natriuretic peptides. **(F)** Meteorin-like. **(G–I)** BMP7. ^&^*P* < 0.05 (Tukey *post hoc* after group × time interaction in ANOVA), difference between sessions in moment 30′. ^χ^ *P* < 0.05 (Tukey *post hoc* after group × time interaction in ANOVA), difference between sessions in moment 60′. ^#^*P* < 0.05 (Tukey *post hoc* after group × time interaction in ANOVA), difference to moments pre and post of pre training session. *P < 0.05 (dependent *t*-test), difference between sessions in area under curve (AUC). *n* = 15 per group, except FNDC5/irisin *n* = 8; and natriuretic peptides *n* = 12, both in feeding group. Values are in mean.

Results are presented as means ± standard deviation (Tables 2, 3) or mean ± standard error (Figures 3, 4) or mean (Figure 5 – AUC). The level of significance used was *P* < 0.05. All analyses were performed using Statistica 6.0 software.

**TABLE 2:** Clinical parameters of volunteers.

	Pre	Post	*P*
Female/male	9/6	−	−
T2D diagnosis (years)	5.56 ± 2.55	−	−
Age (years)	50.67 ± 3.97	−	−
Height (cm)	1.67 ± 0.07	−	−
Weight (kg)	83.62 ± 13.41	83.05 ± 13.41	0.08
BMI (kg/m^2^)	29.61 ± 3.60	29.41 ± 3.61	0.08
Neck circumference (cm)	39.63 ± 4.47	38.83 ± 4.50^a^	<0.0001
Waist circumference (cm)	96.86 ± 10.20	94.33 ± 10.04^a^	<0.0001
Hip circumference (cm)	106.26 ± 6.22	104.46 ± 5.82^a^	0.01
Lean mass (kg)	52.94 ± 10.00	54.12 ± 10.90^a^	<0.001
Fat mass (%)	35.79 ± 5.21	34.37 ± 5.41^a^	<0.001
VO_2_max. (ml/kg/min)	21.48 ± 4.06	23.59 ± 4.21^a^	<0.01
Leg press 1 RM (kg)	194.80 ± 78.22	245.33 ± 88.62^a^	<0.0001
Bench press 1RM (kg)	33.73 ± 23.27	40.60 ± 22.73^a^	0.02
RMR (kcal/day)	1328.21 ± 347.16	1442.09 ± 396.96	0.08
Systolic BP (mmHg)	117.30 ± 14.40	113.10 ± 14.70	0.14
Diastolic BP (mmHg)	74.40 ± 11.20	73.20 ± 9.00	0.17
Resting heart rate (bpm)	81.26 ± 11.87	75.26 ± 11.13^a^	0.03
Carbohydrate consumption (g/day)	311.80 ± 95.03	318.15 ± 93.03	0.77
Lipid consumption (g/day)	55.30 ± 20.63	54.14 ± 16.58	0.77
Protein consumption (g/day)	71.2 ± 21.82	79.55 ± 21.63	0.36
Total calories (kcal/day)	1661.28 ± 473.13	1713.11 ± 483.98	0.97
Erythrocytes (mi/mm^3^)	4.81 ± 0.48	4.90 ± 0.37	0.08
Hemoglobin (g/dl)	13.7 ± 1.19	14.06 ± 1.15^a^	0.04
Hematocrit (%)	40.26 ± 2.86	41.71 ± 2.31^a^	0.01
C peptide (ng/ml)	2.68 ± 1.07	2.38 ± 0.71	0.25
Insulin (μU/ml)	14.25 ± 7.93	12.98 ± 6.09	0.11
Glucose (mg/dl)	141.40 ± 34.34	121.20 ± 14.25^a^	0.02
HbA1c in mmol/mol/(%)	63.5/(7.96 ± 2.42)	55.2/(7.20 ± 1.55)^a^	0.04
HOMA-beta (%)	72.86 ± 44.71	88.13 ± 62.18	0.23
HOMA-IR (%)	5.33 ± 3.80	4.24 ± 2.24	0.08
Triglycerides (mg/dL)	158.13 ± 82.53	125.66 ± 46.49^a^	0.04
Total cholesterol (mg/dL)	179.33 ± 35.17	181.20 ± 35.89	0.82
HDL-c (mg/dL)	41.06 ± 16.11	43.33 ± 18.28^a^	0.04
LDL-c (mg/dL)	105.40 ± 24.02	106.73 ± 20.78	0.91
VLDL-c (mg/dL)	31.66 ± 16.47	23.93 ± 8.71^a^	0.02
TSH (IU/ml)	1.26 ± 2.990.56	1.47 ± 0.65	0.18

*BMI, body mass index; VO_2_max, maximum oxygen consumption; 1RM, one maximum repetition; BP, blood pressure; HbA1c, glycated hemoglobin; HDL-c, high-density lipoprotein cholesterol; HOMA-IR, homeostasis model assessment-insulin resistance; LDL-c, low-density lipoprotein cholesterol; RMR, resting metabolic rate; T2D, type 2 diabetes mellitus; TSH, thyroid-stimulating hormone; VLDL-c, very-low density lipoprotein cholesterol.*

*^a^P < 0.05, t-test dependent samples. Values are in mean ± standard deviation.*

**TABLE 3:** Serum thermogenesis inductors in the fasting and fed state before training.

	Pre fast	Post fast	Pre fed	Post fed
IL4 (pg/ml)	2.68 ± 1.91*	2.71 ± 1.71^#^	4.61 ± 1.57	4.83 ± 1.22
IL13 (pg/ml)	72.35 ± 16.67	63.85 ± 15.01^#^	505.18 ± 821.45	515.44 ± 756.83
IL33 (pg/ml)	10.86 ± 5.14*	12.17 ± 5.17^a#&^	15.97 ± 9.33	14.32 ± 8.53
BMP4 (pg/ml)	19.59 ± 19.45	25.40 ± 28.24	22.08 ± 13.93	21.81 ± 9.06
BMP7 (pg/ml)	22.57 ± 21.83	21.88 ± 17.02	17.72 ± 18.56	18.05 ± 24.67
FGF21 (pg/ml)	145.32 ± 170.36	139.25 ± 172.59	187.70 ± 230.38	169.80 ± 198.09
NAT. PEP. (ng/ml)	3.23 ± 3.42*	3.83 ± 4.27^#^	0.27 ± 0.12	0.23 ± 0.11
Meteorin-like (pg/ml)	330.84 ± 77.29*	329.54 ± 80.16^#^	90.34 ± 59.88	94.62 ± 47.57
FNDC5/irisin (μg/ml)	1.30 ± 0.64*	1.63 ± 0.71^a#^	0.80 ± 0.66	1.19 ± 1.10

*IL, interleukin; BMP, bone morphogenic protein; FGF, fibroblast growth factor; NAT. PEP., natriuretic peptides.*

*^a^P < 0.05, dependent t-test pre versus post.*

^*^P < 0.05, dependent t-test fed versus fasting state in pre-training.

*^#^P < 0.05, dependent t-test, fed versus fasting state in post-training.*

*^&^P ≤ 0.05, ANCOVA, difference pre–post response between fasting and feeding. n = 15, except FNDC5/irisin n = 8 and NAT. PEP. n = 12 in fed. Values are in mean ± standard deviation.*

## Results

### Combined Training Improved Clinical Markers and Metabolic Status

As expected, CT induces improvement in several clinical variables associated with body composition and biochemical markers related to glycemic and lipid metabolism. CT protocol has reduced neck, waist, and hip circumferences, fat mass, resting heart rate, glucose, glycated hemoglobin, triglycerides, and VLDL-c. Furthermore, CT increased hemoglobin, Hematocrit, lean mass, muscle strength (leg press and bench press exercises), VO_2_max, and HDL-c (Table 2). No changes were observed in food behavior (Table 2) and white blood cells (data not shown).

It is important to note that these results (except blood cell count) were presented in a primary randomized controlled trial (Bonfante et al., under review). In a group/time comparison design, the results are the same as here observed for neck and waist circumferences, fat mass, resting heart rate, glucose, glycated hemoglobin, triglycerides, VLDL-c, lean mass, muscle strength, and VO_2_max.

### Combined Training Effects on Pro-thermogenic/Anti-inflammatory Markers Concentrations

Combined training increased pre- and post-training baseline IL33 (*P* = 0.01) and FNDC5/irisin (*P* = 0.04) on fast (Table 3). No changes were observed in pre- and post-training fed (Table 3). In a group/time comparison design of the primary study, IL33 and FNDC5/irisin presented the same pattern of behavior in the comparison between Δ% of the training and CG.

Because of these CT effects on fasting pro-thermogenic/anti-inflammatory markers concentrations and clinical/metabolic status, we performed correlations between the Δ% of changing of these variables. As a result, positive correlations were observed between IL-4/eosinophils; IL-13/lymphocytes (*r* = 0.79, *P* < 0.01); FNDC5-irisin/HDL-c (*r* = 0.83, *P* = 0.02); meteorin-like/basophils (*r* = 0.45, *P* = 0.04) and between bench press (*r* = 0.53, *P* = 0.04); FGF21/VO_2_max (*r* = 0.63, *P* = 0.01) and leg press (*r* = 0.63, *P* = 0.01); and BMP7/eosinophils (*r* = 0.81, *P* = 0.02). Negative correlations were also observed between IL4/hip circumference (*r* = −0.62, *P* = 0.01); IL13/glucose (*r* = −0.78, *P* = 0.03); FNDC5-irisin/resting heart rate (*r* = −0.64, *P* < 0.01); meteorin-like/diastolic blood pressure (*r* = −0.53, *P* = 0.04), neck circumference (*r* = −0.53, *P* = 0.04); FGF21/insulin (*r* = −0.60, *P* = 0.01), C peptide (*r* = −0.54, *P* = 0.03); and BMP4/waist circumference (*r* = −0.81, *P* = 0.02).

### Baseline Fasting/Fed Pro-thermogenic/Anti-inflammatory Markers Concentration

The data about serum thermogenesis inductors in the fasting and fed states before training are shown in Table 3. IL4 (*P* = 0.01) and IL33 (*P* = 0.02) exhibited a decrease in fasting when compared to feeding, while natriuretic peptides (*P* = 0.01), meteorin-like (*P* < 0.001), and FNDC5/irisin (*P* = 0.01) exhibited an increase in fasting (Table 3).

### Interactions Between Training and Fasting/Fed on Pro-thermogenic/Anti-inflammatory Markers Concentration

After training, IL4 (*P* = 0.001), IL13 (*P* = 0.02), and IL33 (*P* = 0.05) presented a decrease in fasting when compared to feeding, while natriuretic peptides (*P* < 0.01), meteorin-like (*P* < 0.001), and FNDC5/irisin (*P* = 0.04) remained increased in fasting (Table 3).

In pre/post responses between fasting and fed status (conditions × time comparison) were observed differences only for IL33 (Table 3), probably occurred due to the increase observed between pre- and post-training fasting.

### Effects of the First and Last Training Session: Sedentary Versus Trained Status

Based on evidence that exhibits the acute secretion of pro-thermogenic/anti-inflammatory inducers by exercise, as well as the differences in the responses according to the subjects’ physical fitness, we analyzed the time-course serum responses of these inductors’ outcomes in the first and last training session. In the first training session IL4 (*P* = 0.03), IL13 (*P* = 0.02), and IL33 (*P* = 0.03) show a decrease in the post 60′ when compared to the pre-training moment in the pre-session (Figures 3A–C). In FNDC5/irisin, we observed an increase in the post 60′ moment, when compared to the pre (*P* = 0.04) and post 30′ (*P* = 0.03) moments in the first-training session (Figure 3D). Natriuretic peptides were increased in the post 30′ (*P* < 0.01) moment when compared to the pre-moment in the first training session (Figure 3E). For meteorin-like, there was a decrease in the post 30′ (*P* = 0.01) when compared to post moment (Figure 3F). No significant changes were observed for FGF21, BMP4, and BMP7 in the first session (Figures 3G–I).

The results of the last training session are shown in Figure 4. There was an FNDC5/irisin decrease in the post 60′ compared to the post (*P* < 0.01) and post 30′ (*P* < 0.01) moments (Figure 4D). Natriuretic peptides were increased in the post 60′ moment when compared with the pre (*P* = 0.01) and post 30′ (*P* = 0.01) moments (Figure 4E). Again there was a decrease in meteorin-like, but this time at the post 60′ moment (*P* = 0.01) when compared to the post moment (Figure 4F). BMP4 exhibited an increase in the post moment when compared to the pre moment (*P* < 0.01) in the post-training session (Figure 4H). No significant changes were observed for IL4, IL13, IL33, FGF21, and BMP7 in the last session (Figures 4A–C,G,I).

The differences between first and last time-course sessions (sessions × time and AUC) are shown in Figure 5. IL4 (*P* = 0.04) (Figure 5A) and Natriuretic peptides (*P* = 0.01) (Figure 5E) presented differences in post 60′ moment between sessions, while this difference was observed for IL33 in post 30′ moment (*P* = 0.04) (Figure 5C). Natriuretic peptides showed also difference in last session post 60′ to moments pre (*P* = 0.01) and post (*P* = 0.01) of pre-training session (Figure 5E). Different AUCs were observed between sessions in FNDC5/irisin, natriuretic peptides, and meteorin-like (Figures 5D–F). No significant changes were observed for IL4, IL13, IL33, FGF21, BMP4, and BMP7 between the first and last sessions (Figures 5A–C,G–I).

## Discussion

In the present study, we evaluated the chronic and acute training responses and the fed/fasted state of serum pro-thermogenic/anti-inflammatory inducers in overweight type 2 diabetics individuals. We show that adaptation to physical training and a better metabolic pattern favor an improvement in the acute secretory pattern in part of the pro-thermogenic and anti-inflammatory substances. In pre- and post-training, fed and fasting states also interfere differently in these substances, where fasting interferes in the increase of myokines, while the fed state induces an increase in interleukins.

The clinical outcomes support that the other results of the present study after the training period occurred simultaneously with a better metabolic pattern obtained by the CT practice. The CT here applied induced the well-known benefits of strength training (increase in strength and muscle mass) and AT (improvement of aerobic capacity and lipid metabolism, as well as a decrease in body fat and resting heart rate) (Colberg et al., 2010, 2016). In addition, our group observed additional improvements using CT when compared to the strength and AT alone (Libardi et al., 2012), decreased low-grade subclinical inflammation and insulin resistance (Brunelli et al., 2015; Bonfante et al., 2017), interferes in concentrations of certain myokines (Brunelli et al., 2015; Bonfante et al., 2017), and improve global metabolome profile interfering in metabolic pathways related to glucose metabolism, insulin signaling and catecholamines biosynthesis (Duft et al., 2017). Both aerobic and strength training may have influenced the improvement of glycemic metabolism since there is a decrease in glycemic levels and glycated hemoglobin. But, for insulin resistance, only a decreasing trend is observed (*P* = 0.08). This result may have been influenced by the method of analysis (HOMA-IR) or the number of subjects analyzed. Although the HOMA-IR method is widely used to analyze insulin resistance, the gold standard method to evaluate this purpose is the hyperinsulinemic–euglycemic clamp (Kim, 2009). Perhaps, if the clamp method had been used, we could observe insulin resistance results that corroborate with other glycemic metabolism markers here evaluated.

The training effects on the concentrations of IL-33 and FNDC5/irisin can be considered positive due to the metabolic benefits and anti-inflammatory effects of these two substances (Bostrom et al., 2012; Brestoff et al., 2015). Curiously, these differences between pre- and post-training seen in IL33 and FNDC5/irisin occurred only on fasting. This is probably due to the complex interaction between metabolism, energy substrates, hormones involved in the control of hunger/satiety and energy demand, the immune system, and the individuality of the individuals to respond to all these interactions. In fasting, a lower incidence of these interferences and certain patterns of organ responses are more easily observed (Di Francesco et al., 2018; Wallis and Gonzalez, 2019). In addition, the correlation data give evidence of the beneficial associations between these pro-thermogenic/anti-inflammatory substances with metabolic control observed in the literature (Pedersen and Febbraio, 2012), as well as the CT contribution to the modulation of these factors.

In the acute effects of the training session, the interleukin results show that with adaptation to training the serum concentration of these substances tend to maintain and increase sharply after exercise, which is a relevant aspect due to their pro-thermogenic, anti-inflammatory, myogenic differentiation, and growth, and muscle regeneration (Nguyen et al., 2011; Possidonio et al., 2011; Rao et al., 2014; Brestoff et al., 2015; Schiaffino et al., 2017). Meteorin-like is a myokine associated with IL4, IL13, certain lymphocytes, decreased inflammation, and improved insulin resistance (Rao et al., 2014; Li et al., 2015; Jung et al., 2018). Although these associations are not baseline observed, there is a post-training elevation in AUC for this myokine associated with a non-decrease of interleukins.

In the three established myokines analyzed (FNDC5/irisin, meteorin-like, and natriuretics peptides) we have in common the increase of AUC in post-training. Although FNDC5/irisin shows a significant increase only in the first training session and meteorin-like does not have a significant increase in any of the sessions, the AUC increase in post-training indicates a better secretory pattern of these substances in the total time-course analyzed. The enhancement of the natriuretic peptides increase is probably due to the fact that the cardiac musculature is the main secretion site of this peptide (Ramos et al., 2015). The beneficial cardiovascular adaptations traditionally provided by CT, such as increased left ventricular ejection volume and, consequently, cardiac output must have influenced this result. This idea is supported by previous findings, which show that cardiac output during exercise is related to the secretion of natriuretic peptides (Yoshiga et al., 2019).

The results of BMP4 also have acute peaks of secretion optimized with adaptation to training. Curiously, other studies should focus on the evaluation of BMP4 as a myokine since it is expressed in musculoskeletal tissues areas that suffer secretive influences from muscle contraction induced by physical exercises and its metabolic/molecular effects (Tarantino et al., 2015).

Together, these results of acute secretion of pro-thermogenic and anti-inflammatory inducers show that secretory peaks after the training session present an important role in providing the benefits of physical training. Besides, training adaptations favor optimization in the acute secretory pattern of these substances. In addition to training adaptations, the type of exercises, as well as the metabolic pattern of the population analyzed, can also influence the secretion of substances induced by metabolism (Petriz et al., 2017). Thus, the metabolic improvement observed over 16 weeks of training, may also have influenced the present results of the post-training session.

The time-course chosen to evaluate these acute responses was based on other studies with myokines and pro-thermogenic substances or energy needs related (Pedersen and Febbraio, 2012; Petriz et al., 2017; Fox et al., 2018), which usually end up occurring during or in the minutes following the practice of physical exercise. The use of CT protocol was chosen because it is the type of activity recommended for health promotion in this population, including individuals with T2D (Colberg et al., 2010, 2016). In addition, both endurance and strength exercise can also interfere in peptides secretory tissues (for example muscle and fat cells) (Roca-Rivada et al., 2013; Eckardt et al., 2014). For FNDC5/irisin, for example, endurance exercise seems to be the main stimulator (Bostrom et al., 2012; Lee et al., 2014), although acute resistance exercise also leads to a transient increase in these peptides (Nygaard et al., 2015). Further studies should focus on analyzing the responses of different types of exercise protocols and other time-courses since other protocols may promote different secretory peaks. For example, protocols with high-intensity interval training induce the EPOC effect (excess post-exercise oxygen consumption) and consequent additional energy demand for several hours after practicing a training session (Maehlum et al., 1986).

Some studies have observed that a caloric meal or the postprandial period are factors that promote increased secretion of pro-thermogenic products (Vosselman et al., 2013; Din et al., 2017). This may be indicative of the reason for the increase of IL4, IL13, and IL33 in the postprandial moment.

However, caloric restriction in animal models increases IL4, IL13, and IL33, noradrenaline, and consequent thermogenesis in order to generate lipolysis and supply the calorie deficit (Fabbiano et al., 2016, 2018). A possible explanation for this divergence of results is the studies with food deprivation in animals present more aggressive restrictive strategies, differently from the present study. Another aspect that may be related to the decrease of these interleukins, is the fact that fasting induces an increase in cortisol, which influences a functional and secretory decrease of immune system components (Nakamura et al., 2016).

The indication of the increase of the thermogenic inducers natriuretic peptides, meteorin-like, and FNDC5/irisin by fasting may be related to thermal adaptations to compensate for the period without food since there is a certain thermal effect provided by food intake (Brown et al., 2015). Curiously, prolonged fasting in animal models also increased serum concentrations of certain myokines, for example, FNDC5/irisin (Roca-Rivada et al., 2013).

All this context involving serum pro-thermogenic/anti-inflammatory inducers by the effects of exercise and fasting/fed is important due to the association of high concentrations with metabolic health. FNDC5/irisin and natriuretic peptides, for example, have exhibited a decrease in T2D subjects when compared to healthy individuals (Du et al., 2016; Verboven et al., 2017). In addition, primary results of the present research project showed type 2 diabetics individuals with smaller IL4, IL13, IL33, meteorin-like and BMP4 levels when compared to healthy individuals (Bonfante et al., in review). Interestingly, these differences are not observed after training for IL4, meteorin-like, and IL4. Thus, non-pharmacological strategies as physical exercise or food behavior to induce higher concentrations of pro-thermogenic/anti-inflammatory are important, since the increased activity of thermogenic and anti-inflammatory tissues, such as brown adipose tissue, is associated with protection against several cardiometabolic diseases (Becher et al., 2021).

The present study has some limitations, such as comparisons between fasted and fed states on different days. However, certain procedures were taken to minimize this, such as standardized meals and times, assessments performed on the same day of the week, and a short period between assessments. The lack of a CG in the baseline chronic outcomes is also a limitation, but this was the main objective of the primary study of the present research project. Importantly, the results of this primary study (randomized and controlled) corroborate the chronic baseline results found in the present study. Although the number of subjects and statistical power had an adequate standard, probably a larger sample of subjects could support that results with a tendency toward statistical significance (*P* between 0.05 and 0.08) become significant (*P* < 0.05), especially in the case of the technical limitation, which decreased the number of analyzed samples of FNDC5/irisin and natriuretic peptides. Finally, despite the HOMA-IR method being widely used to analyze insulin resistance, not having used the hyperinsulinemic–euglycemic clamp for this purpose also is a limitation.

In conclusion, adaptation to physical training and a better metabolic pattern promote an improvement in the acute secretory pattern in part of pro-thermogenic and anti-inflammatory substances, which should contribute to the metabolic benefits achieved by incorporating an active lifestyle in overweight individuals with T2D. Baseline analyses showed that only part of these substances indicates an increase (IL33 and FNDC5/irisin), although important correlations were observed between these substances with several analyzed clinical variables.

The fed and fasting states also interfere differently in part of these pro-thermogenic and anti-inflammatory substances, where fasting interferes in the increase of myokines, while the fed state induces an increase in interleukins.

## Data Availability Statement

The raw data supporting the conclusions of this article will be made available by the authors, without undue reservation.

## Ethics Statement

The studies involving human participants were reviewed and approved by https://www.avivasysbio.com/. The patients/participants provided their written informed consent to participate in this study.

## Author Contributions

CC supervised the study. IB, RD, KM, JT, EF, AR, DB, MM, MC-M, LV, and CC performed hypothesis, generation, contributed to the design, data analysis, interpretation of results, and manuscript preparation. IB, RD, KM, JT, EF, AR, and DB conducted the experiments, tests, training sessions, and data analysis. IB was the guarantor of this work and, as such, had full access to all of the data in the study and takes responsibility for the integrity of the data and the accuracy of the data analysis. All authors edited and approved the final manuscript.

## Conflict of Interest

The authors declare that the research was conducted in the absence of any commercial or financial relationships that could be construed as a potential conflict of interest.

## Publisher’s Note

All claims expressed in this article are solely those of the authors and do not necessarily represent those of their affiliated organizations, or those of the publisher, the editors and the reviewers. Any product that may be evaluated in this article, or claim that may be made by its manufacturer, is not guaranteed or endorsed by the publisher.

## Footnotes

1
http://www.ensaiosclinicos.gov.br/rg/RBR-62n5qn/
